# Follow-up of the tumor load in patients with de novo chronic myeloid leukemia and in complete cytogenetic remission treated with imatinib in Colombia

**Published:** 2012-12-30

**Authors:** Gonzalo Guevara, Jaime A González, Diego E Lopera, Manuel González, José D Saavedra, José Fernando Lobaton, Jorge Enrique Duque

**Affiliations:** a Instituto Colombiano de Genética y Oncologia Molecular E-mail: incogem@yahoo.com; b Oncologos de Occidente, Armenia, Colombia. E-mail: jaimegonzalezdiaz@hotmail.com; c Oncologos de Occidente, Manizales, Colombia.; d Instituto Medico de Alta Tecnologia (IMAT), Monteria, Colombia. E-mail: Manuel101@gmail.com; e Clinica Vida, Medellin, Colombia E-mail:isaavedra@une.net.co; f Hospital Militar Central, Bogota, Colombia. E-mail: lobatonjf@yahoo.es; g Oncologos Asociados de Imbanaco, Cali, Colombia. E-mail: jorgeduque@telesat.com.co

**Keywords:** Philadelphia chromosome, imatinib, minimal residual disease, BCR-ABL

## Abstract

**Objective:**

To evaluate the hematological, cytogenetic, and molecular responses in Colombian patients with CML chronic myeloid leukemia (CML) treated with imatinib.

**Methods:**

Two groups of patients, one with the novo diagnostic and another in state of complete cytogenetic remission were followed for 12 months with quantitative PCR evaluations every three months and with chromosomal analysis every 6 months.

**Results:**

The group with the novo diagnosis showed 50% of complete cytogenetic remission at 12 months while the other 50% were considered to have primary resistance. Respect the molecular analysis, 10.5% of the patients reached undetectable BCR-ABL transcripts at 12 months. In the complete cytogenetic remission group, 10.6% lost the state of complete cytogenetic remission at 12 months, 50% reached undetectable BCR-ABL transcripts but 10% showed levels higher than 10%, which in our standardization was equal to no molecular response.

**Conclusions:**

Despite having received the conventional dosages of 400 mg/day of imatinib, the cytogenetic and molecular responses obtained in our group of Colombian patients with CML, were lower than those in other international studies.

## Abstract (es)

**Objetivo:**

Evaluar las respuestas hematológica, citogenética y molecular en pacientes colombianos con leucemia mieloide crónica tratados con imatinib.

**Métodos:**

Dos grupos, uno con diagnóstico de novo y otro en el estado de remisión citogenética completa, se siguieron mediante estudios citogenéticos en médula ósea cada 6 meses y reacción en cadena de la polimerasa cuantitativa (Q-PCR) cada 3 meses.

**Resultados:**

En el grupo de novo, el 50% alcanzó el estado de remisión citogenética completa mientras el otro 50% se consideró con Resistencia primaria. Respecto al análisis molecular, 10.5% mostró transcriptos BCR-ABL indetectables. En el grupo de remisión citogenética completa, 10.6% perdió la condición de remisión citogenética completa, en el 50% los transcriptos BCR-ABL fueron indetectables mientras el 10% mostró niveles por encima del 10% considerado como no respuesta molecular, según nuestra estandarización.

**Conclusión:**

Aunque los pacientes recibieron las dosis convencionales de 400 mg/día de imatinib, las tasas de respuesta citogenética y molecular en los pacientes colombianos fueron menores que las obtenidas en estudios internacionales.

## Introduction

Twelve years after the introduction of Imatinib mesylate as a treatment for chronic myelogenous leukemia (CML), the change in the natural history of the disease is evident, manifested in the increase in survivals (89%, estimated in 5 years) compared to that of prior treatments used (68%-70%). This improvement is due to the fact that Imatinib induces significant remission of the Philadelphia clone, which prevents the disease from progressing into acute phases in 97% of the patients that get to complete cytogenetic remission and 100% for those who also reduce the level of the BCR/ABL transcription in 3 logarithms or more within the first 12 months^1^. Despite these amazing achievements, up to 25% of patients are primary resistant to the drug, and a subgroup of responders, suffer a secondary relapse (1.1%-5.5%) ^1^ ^,^ ^2^. Due to this, the strict follow-up of the tumor load with hematological, cytogenetic and molecular parameters, according to the international definitions of optimal response, suboptimal and failure, it is crucial to make objective therapeutic decisions. The evidence about the therapeutic response to Imatinib comes mainly from clinical trials and follow-ups made to patients in developed countries. In order to contrast with those results, we embarked on a mission with the objective to evaluate the hematological, cytogenetic, and molecular response in a group of de novo patients and another with complete cytogenetic response in a developing country such as Colombia.

## Materials and Methods

### Design study:

This investigation, descriptive in nature, was carried out in Colombia between January of 2008 and December of 2009 with the participation of 6 hematologic centers, and was authorized by a local ethics committee. The therapeutic conducts adopted during the follow-up period depended exclusively and freely on the treating physician according to his/her experience and on the protocols of each hematologic center. The patients were divided in two groups: de novo and complete cytogenetic remission (CCR). The criterion for inclusion in the first group was the possibility of BCR/ABL fusion proven by PCR in peripheral blood in patients who were clinically suspected to have CML, and for the second, confirmation of the complete cytogenetic remission state in the bone marrow; additionally patients had to be over 18 years old and have written consent of their participation. The follow-up was done over one year of medical visits with hemogram and molecular assessments every three months and chromosomes every six months: visit 0 (inclusion), visit 1 (90 days), visit 2 (180 days), visit 3 (270 days) and visit 4 (360 days), with some visits made at intermediate periods if the hematologist considered it necessary: the drug was administered by the Health Sponsor Company (EPS in Spanish) to which the patient was affiliated. For the de novo group the Sokal Index was calculated resorting to www.leukemia-net.org at the time of inclusion and the hematological response estimated by each physician according to conventional criterions^3^. The bone marrow samples (3-5 mL) and peripheral blood (10-20 mL) were remitted to room temperature thus guaranteeing their arrival before the 24-hour mark to a central laboratory where the cytogenetic and molecular studies were performed.

### Cytogenetic analysis:

these analyses were performed on bone marrow samples according to published protocols^4^, at the time of inclusion, 6 and 12 months with a minimal recount of 20 mitosis. Upon arrival, the samples were washed three times with a RPMI 1640 solution and were cultured in 10 ml of the same solution with fetal bovine serum (10%) at 37 oC for 72 hours. The mitosis were obtained immediately upon arrival of the samples and every 24 hours, with the purpose of increasing the probability of obtaining evaluable metaphases. Three milliliters of the cell culture were exposed to 10 uL of colchicine (0.06 ug/mL) for 1 hour. After centrifuging, the supernatant was discarded and the cells were submitted to a hypotonic solution of KCl (0.075 M) for one hour. Finally, the cellular pellet was prefixed and washed five times in a Carnoy fixative (methanol: glacial acetic acid 3:1). The microscope slide with the cellular extension was dried at room temperature for 12 hours to later be dyed in a quinacrine solution (0.05%) for 30 minutes to obtain Q bands. The mitosis images were seen through an Olympus BX40 microscope adapted with a mercury lamp of 100 W and with the BandView (Applied Spectral Imaging) software the mitosis were captured and a karyotype was constructed. The cytogenetic response was defined according to the percentage of Ph+ cells: complete (0% Ph+), partial (1% -35% Ph+), minor (66%-95% Ph+) and no response (>95% Ph+) ^5^.

### Real-time polymerase chain reaction:

The quantification of the BCR-ABL transcription was carried out with a real time PCR (RQ-PCR) using TaqMan method over a Light Cycler 480 platform from Roche. The expression of the beta-glucuronidase (GUS) gene was taken as reference for the normalization of the BCR/ABL p210 transcription expression. The oligonucleotide ENF501 (sense), ENR561 (antisense) and the TaqMan ENP541 probe were used to detect the amplification BCR-ABL^6^. As part of our standardization, the mRNA of 30 cases of CML Ph+ de novo was processed in duplicate with RQ-PCR to obtain a median of the (BCR-ABL)X100/(GUS) rate that was taken as a baseline to transform the percentages obtained with a ten-fold logarithmic base. In our case, the median coincided in 100% so that 10% corresponded to a reduction of 1log, 1% of 2log and 0.1% of 3log. Positive and negative controls were included in all of the reactions and the quality of the mRNA and the efficiency of the reverse transcript were judged by the cycle thresholds (Ct) of each sample, so that a Ct higher than 24 was considered inadequate for the analysis for degradation of the mRNA; in this case, the procedure was repeated from the point of extraction of the mRNA. The sensibility was tested using serial dilutions (10^-1^ a 10^-6)^ of an mRNA (1-5 ug/uL) with Ct < 20. These same curves for BCR-ABL and GUS were used as a reference to calculate the relative concentration of the samples with the software that came with the Light Cycler 480.

### Statistical Analysis:

For the descriptive component, percentages were used in the case of discrete and median variables along with their corresponding statistical dispersion (standard deviation) for the case of the continuous variables. In order to illustrate and analyze the behavior of the laboratory variables throughout the follow-up, two-way charts were used. The relation between the quantitative measures of the tumor load in peripheral blood and bone marrow was done with Pearson product-moment correlation coefficient. All of the statistical analysis procedures were done with a Stata 5(r) program. The percentages on each table correspond to the rate of patients that were evaluated at that moment.

## Results

### Patients:

Twenty-five patients were included in the de novo group and 27 in the CCR group, for a total of 52. At the end of the follow-up, 18 patients remained assessables in the de novo group and 19 in the CCR. The losses of the first group were due to three disease-related deaths, one follow-up loss, two to voluntary withdrawals from the study and one to change to another drug; in the second group, one death after an angioedema not related to the illness, three due to therapy failure, two follow-up losses and two voluntary withdrawals. The median dose of imatinib was 400 mg/day, un patient went up to 600 mg and six to 800 mg; when the study was concluded, 4 patients switched to a second generation tyrosine kinase inhibitor (3 dasatinib y 1 nilotinib). After 12 months, 50% of the patients from the de novo group had treatment failure while in the CCR group 11% relapsed. In Table 1, patient's overall characteristics are shown.

**Table 1:**
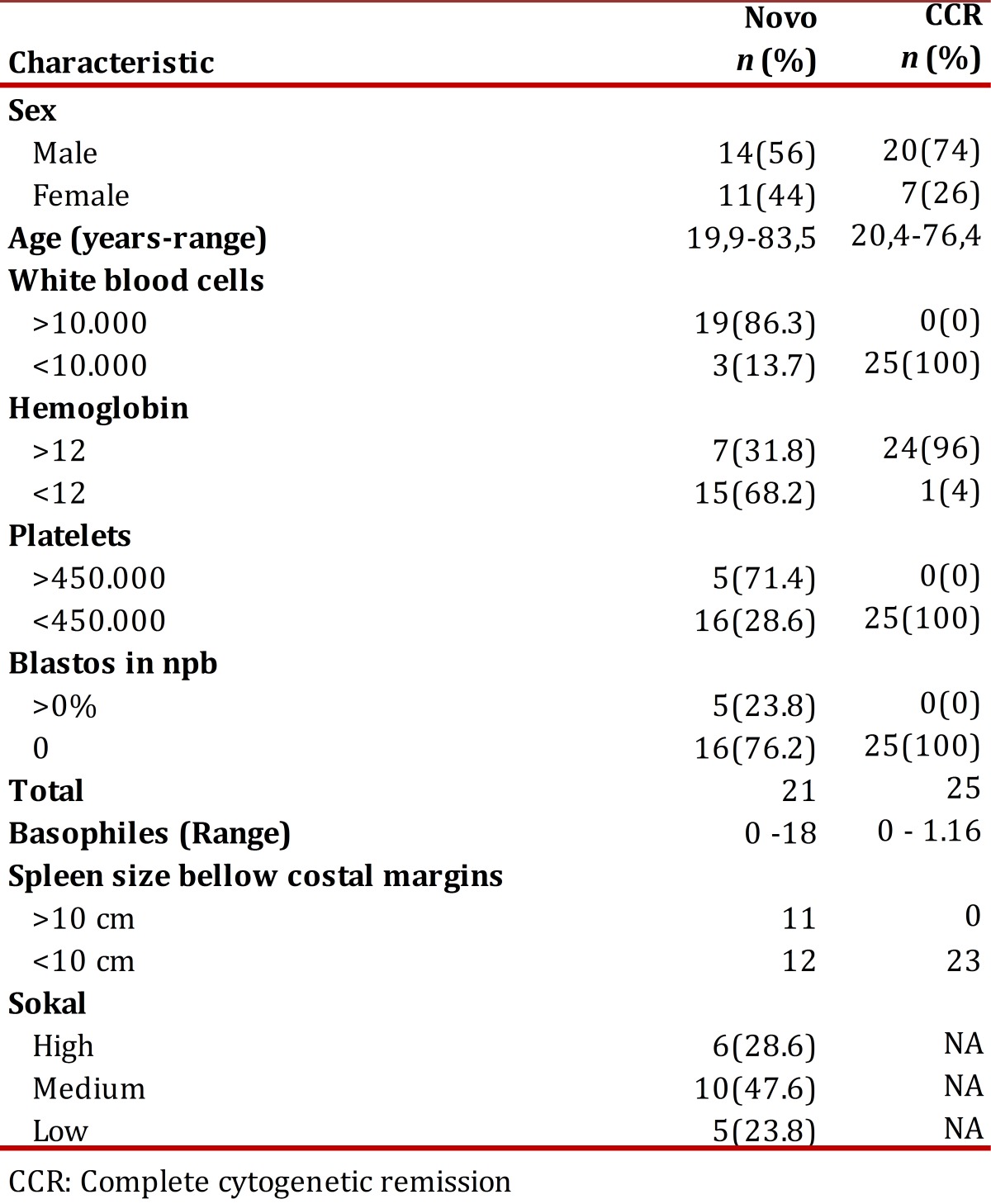
Overall characteristics of the patients at the time of inclusion

### Hematologic response:

**Figure 1:**
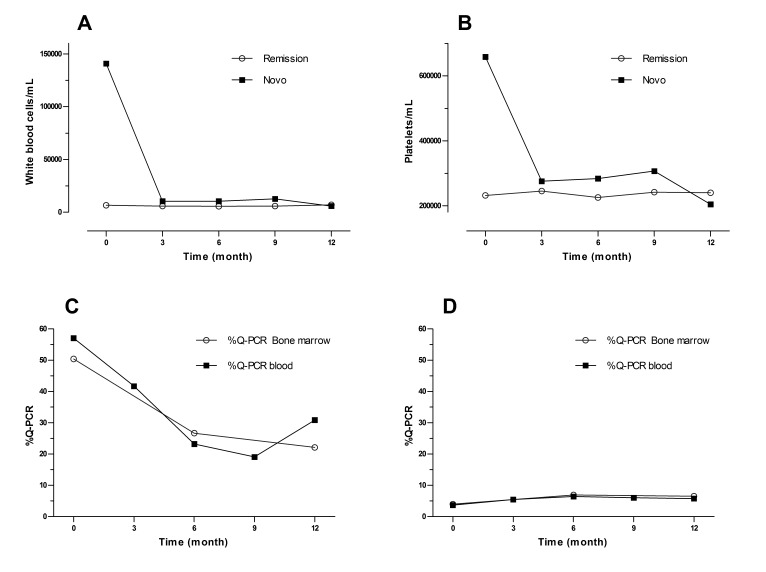
A and B: evolution of the white blood cell and platelets count in both groups ,C and D: correlation between the

The novo group responded quickly to the imatinib with complete responses after 3 months of 79.1%, which increased to 84.2% by the end of the study. In the CCR group after 9 months, 13.6% had lost the hematologic response, although at 12 months all the patients that were evaluated remain in CHR. Figure 1 shows the evolution of the white blood cell and platelets count in the novo group.

### Sokal risk:

In the de novo group, 23.8% remained with low risk, 47.6% remained in medium and 28.6% in high. Table 2 shows the cytogenetic and molecular hematologic response rate according to the Sokal risk in the de novo group at 12 months.

**Table 2:**
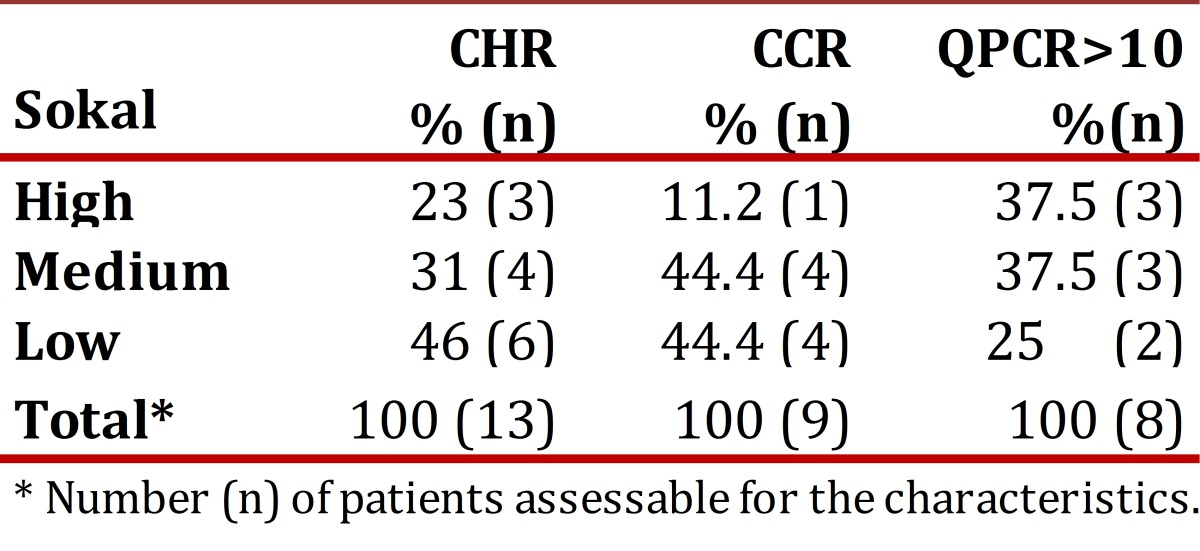
Responses of the de novo group at 12 months according Sokal risk, * Number (n) of patients assessable for the characteristics.

### Cytogenetic response:

Following the 2010 European LeukemiaNet (ELN) criterion, at 6 months 55.5% of the de novo group responded optimally (complete cytogenetic remission plus partial cytogenetic remission), 11% sub-optimal (>35% Ph+) and 33.4% failed (> 95% Ph+). At 12 months, 50% reached an optimal response (complete cytogenetic remission), 5.5% suboptimal (less than complete cytogenetic remission) and 44.5% failed (>35% Ph+) (Table 3). One patient in this group showed clonal evolution with double Ph+ chromosome. In the CCR group, 5 patients (25%) had lost the response after 6 months, 2 had follow-up loss, 2 remained in relapse and 1 recovered complete cytogenetic response at the 12 month control. Therefore, by the end of the follow-up, 89.4% maintained the complete cytogenetic remission state while 10.6% had relapsed (Table 3).

**Table 3:**
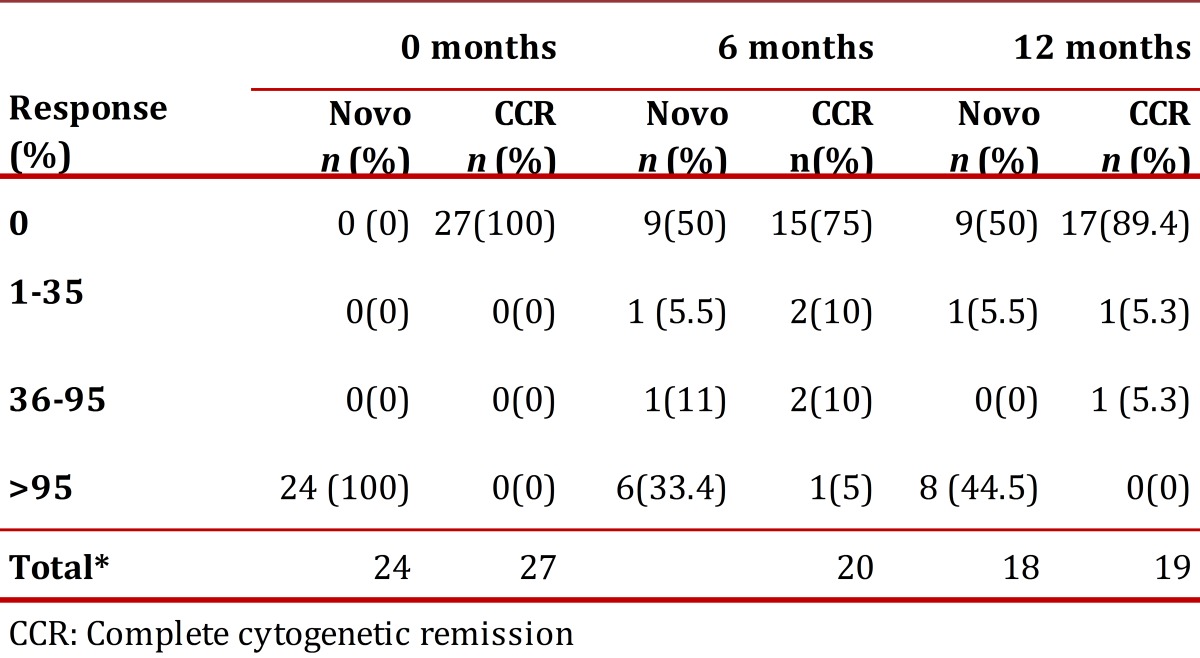
Cytogenetic response in the novo and CCR groups, * Number of patients assessable for the characteristics.

### Molecular response:

In the de novo group a decrement in the transcript BCR-ABL levels during the year of treatment was observed, reaching complete molecular responses (undetectable transcripts) in 5.6% and 11.8% in the bone marrow at 6 and 12 months respectively. These percentages were co-related with the molecular responses obtained in peripheral blood (figure 1). Meanwhile, 53% never decreased their molecular tumor load under 1 logarithm (10%), while 35.2% remained between -1 log (1%) and -3 log (0.1%) (Table 4).

**Table 4.**
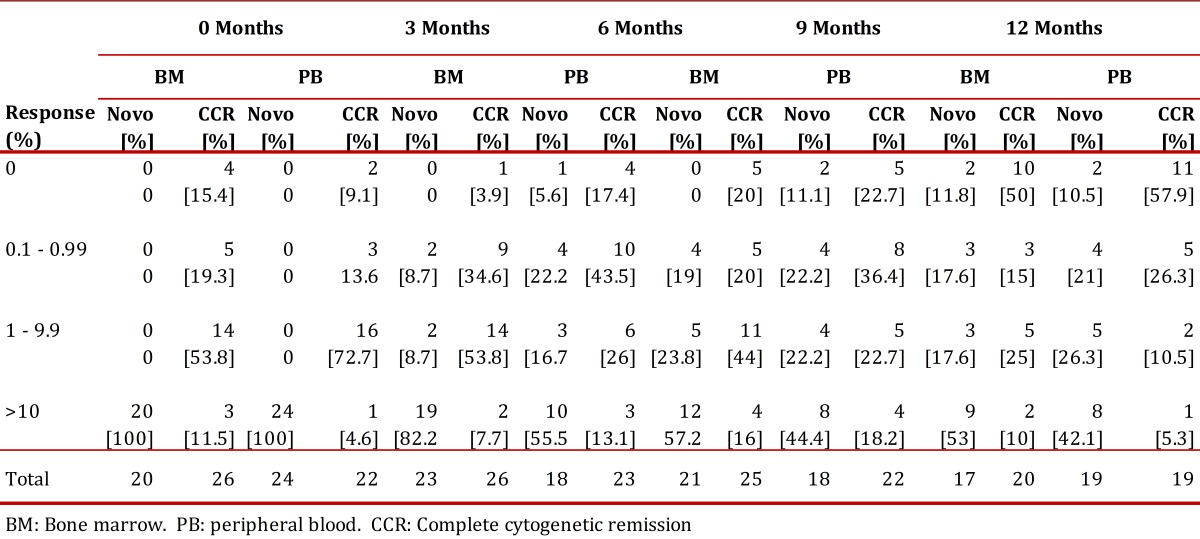
Molecular response in blood and bone marrow in the novo and CCR groups

The complete molecular response in the CCR group was consolidated from 15.4% at inclusion to 50% at 12 months. Three cases (11.5%) showed expressions of the BCR/ABL transcript above 10% (-1 log) at the time of the inclusion, one fell out of follow-up, another exhibited a cytogenetic relapse at 6 months and maintained it until 12 months, and the last held the complete cytogenetic response with decrease of tumor load (Table 4). The correlation between the quantitative measurements of the tumor load in peripheral blood and bone marrow was significant in the respective visits (r=0.72 inclusion visit, r=0.85 visit 2, r=0.66 visit 4) (Figure 1 A and C).

## Discussion

This investigation evaluated the hematological, cytogenetic, and molecular responses in patients with chronic myelogenous leukemia (CML) treated with Imatinib in the context of a developing country where socioeconomic conditions could influence the outcome of biological responses. Thanks to tyrosine kinase inhibitors, which efficiently inhibit the Ph+ clone, the chromosomal and molecular analysis becomes the cornerstone for the monitoring of the tumor load. The biological responses have been synthesized in international guidelines such as ELN which make clinical decision-making much easier^7^. The therapeutic goal according to these guidelines is to reach optimal responses at 3, 6, 12 and 18 months, until complete cytogenetic remissions and major molecular or undetectable transcripts ("complete" remission) can be achieved and sustained over time. It is evident that not every optimal hematological response is accompanied by an optimal cytogenetic response and that complete cytogenetic remission is associated with heterogeneity in molecular response, but this correspondence is 100% in the opposite direction. After a new diagnosis, the first awaited response is the hematological, which in our group was 84.2% at 12 months, lower that those reported in other studies (>96%)^1^ ^,^ ^8^ ^,^ ^9^. The Sokal Index has upheld its predictive relevance regarding complete cytogenetic response: 89% in low, 82% in intermediate and 69% in high, but it loses its significance in the state of complete cytogenetic response^1^. As other authors have observed, our patients with low Sokal Index responded more positively to the hematological, cytogenetic, and molecular criteria, thus reaffirming the importance of defining it at the time of a new diagnosis.

The optimal cytogenetic response at 6 and 12 months was of 55.5% and 50% respectively, inferior to that obtained in clinical trials, which vary from 62% to 69% ^1^ ^,^ ^10^; however, our percentages are higher than those obtained by researchers in other developing countries which report optimal cytogenetic responses for the same periods of between 30% and 49% (MCgR)^11^ ^,^ ^12^. Fifty percent of the novo patients met with the primary refractory criteria for Imatinib due to failed treatments at 6 and 12 months, despite having received the standard dosages or dosages scaled to Imatinib. The probability of obtaining an optimal cytogenetic response from these patients at 18 months is very low. Although not all causes of primary resistance have been established, these include lack of adherence to the treatment, low bioavailability of the drug, pharmacodynamic factors and mutations in the tyrosine kinase region or a combination of the above^13^ ^,^ ^14^. In some essays it has been established that the presences of previous mutations or those occurring within the first months of treatment influence the outcome of treatment response and are associated to a greater probability of progression^15^ ^,^ ^16^. No biological resistance factors were explored in this group of patients but in the future it would be important for all of us to include such analysis as they would allow clinicians to make better decisions regarding the move towards second generation tyrosine kinase. The quantitative molecular evaluations of the BCR-ABL transcripts in real time with PCR are a key part of the CML follow-up, but it requires a highly sensitive, specific, and reproducible test. Our molecular test correspondented well with the cytogenetic response, indicating that it can be used to monitor CML, although it could be improved in regards to some international standards. On the other hand, consensus only exists regarding the intensity of the molecular response at 18 months, when it is considered optimal to reach a decrease equal or below 3 logarithms (RMM) or a suboptimal response with a warning when this response is lost or the levels of the transcript elevate at some point during the treatment^17^.

We implemented a previous local standard for the molecular monitoring establishing a baseline obtained from the Ph+ of 30 de novo patients, which served as a reference in order to transform the percentage of the relation (BCR-ABL)X100/(GUS) of each patient into the respective logarithm. ^18^ ^,^ ^19^ According to this reference, 100% of our novo patients showed expressions greater that 10% (>-1 log) at the time of diagnosis, which dropped 53% (in the bone marrow) by the end of the study and were therefore considered primary resistance patients. The 10% limit (>-1 log) corresponding to the non-hematological response and non-cytogenetic remission states, with low probability of improving their response further on with Imatinib. Meanwhile, 11.8% of the novo patients reached undetectable BCR-ABL transcripts at 12 months, which constitutes a group of low risk of progression. As has been observed in several follow-up studies, all patients with BCR-ABL transcript levels below -3 log and with complete cytogenetic remission do not suffer disease progression, while the same does not occur in the 2% that have higher RMM levels but are still in cytogenetic remission. However, this difference is not considered clinically relevant and therefore the prognostic value of the greater molecular response is still a matter of discussion ^1^ ^10^ ^,^ ^20^ ^,^ ^21^. 47.3% of our patients did not reach MMR state by the end of the study, which should constitute a warning in regards to the strict monitoring of their tumor load. Although the molecular response percentages in this trial are below those obtained in other studies^1^ ^,^ ^10^, it has been established that an important fraction of our patients respond quickly and efficiently to Imatinib, but it will be for further studies to investigate why a greater percentage of molecular response was not obtained.

Of the long-term follow-ups it has been concluded that in order to reach the state of complete cytogenetic remission at 18 months it is necessary to respond optimally with minor cytogenetic remission at 3 months and with partial cytogenetic remission at 6 months, as opposed to patients classified with failure or suboptimal response in these same time periods, which have a low probability of reaching complete cytogenetic remission, and if they do, have a high probability of losing it later on^19^ ^,^ ^22^. More specifically, the accumulated incidence of complete cytogenetic response at 5 years, according to the cytogenetic response at 6 months, was of 98% for those in major cytogenetic remission, 91% for those with minor cytogenetic response, and 25.4% for those who had no response^10^.

Although the majority of the patients continue with the course of treatment for a long period after reaching the state of complete cytogenetic remission, there is still a subgroup that loses it, (with accumulative incidence of 20% to 26% on 4 years) (between 20% and 26%^23^ ^,^ ^24^, with an accumulated incidence of 4 years). The loss of cytogenetic response in our CCR group, for which the inclusion criteria was sate of complete cytogenetic remission, occurred in 5 (25%) patients at 6 months; two of these remained in relapse and one regained complete remission at 12 months, so in the end, 10.6% lost cytogenetic response. These percentages, given the briefness of the monitoring period, are much higher than those reported in other trials, although we were not aware of how long they had been in cytogenetic remission prior to the beginning of the study.

Today there is evidence that one of the main causes of relapse is the emergence of genetically unstable clones refractory to imatinib due to mutations in the BCR-ABL kinase domain or carries of additional cytogenetic anomalies to Ph+; the first was not evaluated in this study and the second was not observed in our patients. Contrariwise, the molecular response observed in the CCR group at the time of inclusion was heterogeneous (Table 4) with 11.5% expressing levels above 10%, which constitutes a risk for future cytogenetic relapse. According to this statement, it has been reported that in order to sustain complete cytogenetic response for a prolonged period of time it is a requisite to obtain a major molecular response at the time of reaching complete cytogenetic remission^25^. During follow-up, 50% of the CCR patients consolidated complete molecular responses (undetectable transcripts), making them a low risk for progression. These observations highlight the importance of incentivizing molecular monitoring once the com.

This investigation intended to evaluate the tumor load in a series of patients undergoing treatment with Imatinib without interfering with the therapeutic decisions. The important limitation of this study derive from this observational design, such as being unable to control the variables that influence in the responses like the adherence to treatment, undefined criteria used to escalate the dosage of Imatinib, among other things. Also, the small and restricted size of the group and loss of patients limited our use of statistics in order to further inquire about significant differences between categories. In conclusion, the investigation showed that the optimal cytogenetic responses expected in the novo group and the losses in hematological and cytogenetic responses in the CCR group are not similar to those obtained in clinical trials and reported in developed countries. It is important to know whether similar findings occur in other developing countries and we must call attention to search social, biological and clinical-management reasons that may hinder better outcomes. One measure to be taken would be to adhere to international response criteria with greater rigor and we hope the approval of second-generation tyrosine kinase inhibitors as a first line treatment may improve response rates in our medium.
